# Determination of Early and Late Endothelial Progenitor Cells in Peripheral Circulation and Their Clinical Association with Coronary Artery Disease

**DOI:** 10.1155/2015/674213

**Published:** 2015-09-16

**Authors:** Shotoku Tagawa, Chiaki Nakanishi, Masayuki Mori, Tsuyoshi Yoshimuta, Shohei Yoshida, Masaya Shimojima, Junichiro Yokawa, Masa-aki Kawashiri, Masakazu Yamagishi, Kenshi Hayashi

**Affiliations:** Division of Cardiovascular Medicine, Kanazawa University Graduate School of Medicine, 13-1 Takara-machi, Kanazawa 920-8641, Japan

## Abstract

The clinical implications of early and late endothelial progenitor cells (EPCs) in coronary artery disease (CAD) remain unclear. We investigated endothelial dysfunction in CAD by simultaneously examining early and late EPC colony formation and gene expression of specific surface markers in EPCs. EPCs were extracted from a total of 83 subjects with (*n* = 47) and without (*n* = 36) CAD. Early and late EPC colonies were formed from mononuclear cells extracted from peripheral blood. We found that fewer early EPC colonies were produced in the CAD group (7.2 ± 3.l/well) than those in the control group (12.4 ± 1.4/well, *p* < 0.05), and more late EPC colonies were produced in the CAD group (0.8 ± 0.2/well) than those in the control group (0.25 ± 0.02/well, *p* < 0.05). In the CAD group, the relative expression of CD31 and KDR of early and late EPCs was lower than in the control group. These results demonstrate that CAD patients could have increased late EPC density and that early and late EPCs in CAD patients exhibited immature endothelial characteristics. We suggest that changes in EPC colony count and gene expression of endothelial markers may have relation with development of CAD.

## 1. Introduction

Endothelial progenitor cells (EPCs) are of interest to medical researchers because of their role in the pathogenesis of atherosclerotic diseases such as coronary artery disease (CAD) [1, 2]. Previous studies have shown that the number of EPCs is related to vascular repair and endothelial restoration and that EPC levels decrease in patients with diabetes mellitus, hypertension, and CAD [2–4]. Recently, EPCs have been obtained from mononuclear cells (MNCs) and studied as two types of culture period-dependent cells. Early EPCs, which produce colonies consisting of spindle-shaped cells in the early period of culture (several days), have limited proliferative capacity, and late EPCs, which produce colonies in the later period of culture (3-4 weeks), have a distinctively strong growth capacity [5–7].

EPCs can be considered as proliferative cells equipped with endothelial property, and both EPC types express cell surface markers specific to endothelial cells. However, EPCs remain difficult to isolate from peripheral blood because they are very rare in peripheral circulation, and therefore, a definition for EPCs is not yet available [1, 5]. EPCs originate from hemangioblast cells in the bone marrow and are differentiated into endothelial cells. Circulating EPCs are recruited to endothelial tissues suffering from hypoxia, and they attend to blood vessel formation and repair in affected tissues [5]. They can be identified by cellular morphology, including specific cell surface proteins, and by examining the expression of genes encoding surface markers [8–10]. In this study, we tested the hypothesis that endothelial dysfunction and impaired endothelial repair in atherosclerotic disease are due to impaired EPCs, which are disturbed during maturation and, thus, prevented from becoming mature and effective EPCs. Specifically, we studied the characteristic features of early and late EPCs in the presence or absence of CAD by simultaneously examining colony formation in vitro and the gene expression of specific surface markers.

## 2. Materials and Methods

### 2.1. Patients and Control Subjects

The local ethics committee of the Kanazawa University Graduate School of Medicine approved the use of human cells in this study, and informed consent was obtained from all individual donors. All CAD patients underwent a coronary angiography and exhibited significant stenosis of the coronary arteries (significant stenosis was defined as >70% diameter stenosis, according to AHA guidelines). Control subjects consisted of persons without organic stenosis in their coronary arteries and healthy volunteers free from clinical symptoms such as angina and cardiovascular risk factors.

### 2.2. EPC Isolation and Culture

Circulating EPCs were isolated from 30 mL of heparinized peripheral blood collected from donors. Briefly, MNCs were isolated using Ficoll-Paque PLUS (GE Healthcare, Waukesha, USA) density gradient centrifugation (400 ×g, 20°C, 20 min), washed twice with phosphate-buffered saline (PBS), counted, and suspended in PBS. MNCs from each donor were cultured separately in an incubator on 6-well cell culture plates coated with fibronectin (BD Biosciences, Heidelberg, Germany) at a density of 5 × 10^6^ cells/well.

Early EPCs and late EPCs were cultured according to the following protocols.

For early EPCs, MNCs were cultured for 10 days in *α*-MEM (Gibco-Life Technologies, Karlsruhe, Germany) with 20% fetal bovine serum (FBS) (Gibco-Life Technologies). For late EPCs, MNCs were cultured for 28 days in complete EGM-2 culture medium supplemented with EGM-2MV (Lonza, Basel, Switzerland). The media were changed three times per week [1, 11].

### 2.3. Enumeration of Early and Late EPC Colonies

The early and late EPC colonies were counted using phase contrast microscopy with a Diaphot microscope (Nikon, Tokyo, Japan), according to the following methods. For early EPCs, the number of colonies was counted in four randomly chosen fields per well at ×40 magnification; for late EPCs, all colonies per well were counted [1]. As for variability of colony count, intraobserver variability was <2.7% and interobserver one was <5%.

### 2.4. Immunofluorescent Staining

Early and late EPC colonies were prepared for the lectin binding and uptake studies using the following methods. First, EPCs were incubated with 1,1′-dioctadecyl-3,3,3′,3′-tetramethyindocarbocyanide-labeled acetylated low-density lipoprotein (Dil-Ac-LDL; Biomedical Technologies Inc., Stoughton, USA) at 10 *μ*g/mL in EGM-2 medium at 37°C for 4 h. They were then washed three times with PBS and fixed with 4% formaldehyde in PBS for 20 min at room temperature. Subsequently, the EPCs were incubated with 200 *μ*L of mouse anti-human UEA-1 (Ulex lectin) antibody-conjugated with fluorescein isothiocyanate (FITC) (Sigma, St. Louis, USA) at 4°C for 1 h and then washed three times with PBS.

Otherwise, early and late EPCs were washed twice with PBS and fixed with 2% paraformaldehyde in PBS and then incubated for 18 h at 4°C in the dark with the following antibodies: a phycoerythrin- (PE-) conjugated anti-human CD34 antibody (BD Bioscience, USA), a FITC-conjugated anti-human CD45 antibody, and a PE-conjugated anti-kinase domain receptor (KDR) antibody (R&D Systems, USA). Stained colony dishes were observed with an immunofluorescence microscope (Keyence, Osaka, Japan).

### 2.5. Quantitative Reverse Transcription-Polymerase Chain Reaction (qRT-PCR)

We measured the mRNA levels of surface markers by using qRT-PCR. Adherent EPCs that formed colonies were harvested mechanically with cell scrapers. Total RNA was extracted from the harvested EPCs using the RNeasy Mini Kit (Qiagen, Venlo, Netherlands) according to the manufacturer's instructions. The extracted RNA (500 ng) was subjected to cDNA synthesis using the Super Script III First-Strand Synthesis System (Invitrogen, CA, USA) in a final volume of 20 *μ*L. An ABI PRISM 7000 sequence detection system (Applied Biosystems, CA, USA) was used to perform quantitative assessment of RNA levels. Glyceraldehyde 3-phosphate dehydrogenase (GAPDH) was used as a control for reactions. The primer sequences of surface markers used for qRT-PCR were as follows: CD45 (forward, 5′-TGGGACTGCTGAGAAGTGCA-3′; reverse, 5′-GGCCCGGGAGGTTTTCATT-3′), CD31 (forward, 5′-TGTATTTCAAGACCTCTGTGCACTT-3′; reverse, 5′-TTAGCCTGAGGAATTGCTGTGTT-3′), KDR (forward, 5′-TACAGACCCGGCCAAACAA-3′; reverse, 5′-TTTCCCCCCTGGAAATCCT-3′), and GAPDH (forward, 5′-GGTGGTCTCCTCTGACTTCAACA-3′; reverse, 5′-GTGGTCGTTGAGGGCAATG-3′). The results of qRT-PCR were recorded as CT values (threshold cycle), which were defined as the cycle number at which a fluorescent signal was generated by SYBR Green I (Applied Biosystems, CA, USA). In data analysis, the mRNA levels of a given gene were evaluated by using the ΔΔCT method.

### 2.6. Statistical Analyses

Data were expressed as the mean ± SEM. Data were analyzed by using ANOVA, with subsequent comparisons by Student's *t*-test. Statistical significance was defined as *p* < 0.05.

## 3. Results

### 3.1. Subject Profiles

The patient and control subject details are shown in Table 1. Eighty-three individuals were enrolled consisting of 47 patients with CAD and 36 controls. Coronary angiography was conducted in all of the CAD patients and 12 controls. Patients had various cardiovascular risk factors such as diabetes, hypertension, and/or dyslipidemia and had been administered medications corresponding to each disease. However, laboratory data showed that there were no significant differences in the levels of malondialdehyde-modified low-density lipoprotein/low-density lipoprotein-cholesterol, high sensitivity C-reactive protein, and hemoglobin A1c between the CAD and control groups.

### 3.2. Morphology and Immunofluorescent Staining of Early and Late EPC Colonies

**Figure 1:**
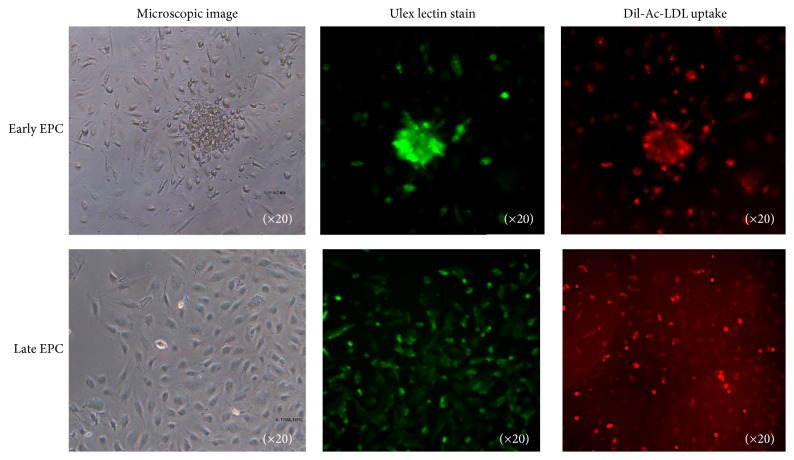
Morphology and immunofluorescence staining of early and late EPCs. Upper panels show images of early EPCs and lower panels show those of late EPCs. Left-hand panels show phase contrast microscopy images, middle panels show images of EPCs stained for Ulex lectin, and right-hand panels show images of Dil-Ac-LDL uptake.

Early EPC colonies characterized by discrete cell aggregates were observed 4–6 days after MNCs were initially seeded, and these colonies were counted 10 days after seeding (Figure 1). Late EPC colonies were observed approximately 2–4 weeks after seeding. First, small colonies appeared, and then cells eventually proliferated to form a monolayer tissue with cobblestone morphology similar to that of the native endothelium (Figure 1). The late EPC colonies were counted 28 days after seeding.

We stained EPC colonies with several antibodies to identify their characteristic surface markers. However, because it is currently difficult to identify EPCs by staining with a single surface marker, staining with a combination of surface markers was necessary (e.g., the combination of CD34 and KDR or FITC-UEA-1 lectin and uptake of Dil-Ac-LDL). As described in a previous study [4], we examined colonies of early and late EPCs that were double positive for staining with UEA-1 lectin and Dil Ac-LDL (Figure 1).

**Figure 2:**
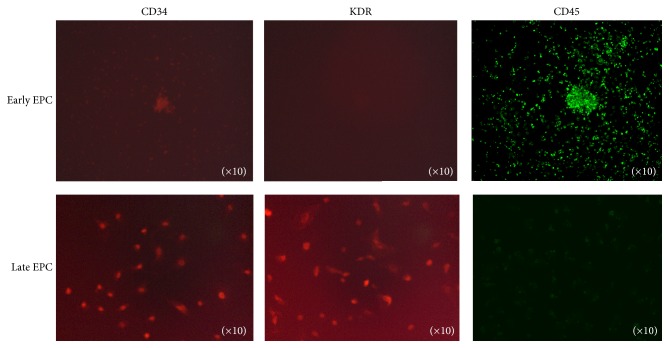
Immunofluorescence detection of anti-CD34, anti-KDR, and anti-CD45 antibodies. Immunofluorescence staining of early and late EPCs is shown in the upper and lower panels, respectively. Left-hand, middle, and right-hand panels demonstrate staining for CD34, KDR, and CD45, respectively. The original magnification was ×10.

Immunofluorescent staining by anti-CD34 and anti-KDR antibody showed that early EPCs were positive for CD34 but negative for KDR (Figure 2). Similarly, late EPCs were stained positive for anti-CD34 and anti-KDR antibody. Importantly, early EPCs were stained positive for leukocyte antigen CD45, whereas late EPCs were stained negative (Figure 2). Based on these immunoreaction results, a combination of the surface markers was used to identify EPC types in the following experiments.

### 3.3. Population Levels and Surface Markers of Early and Late EPC Colonies

**Figure 3:**
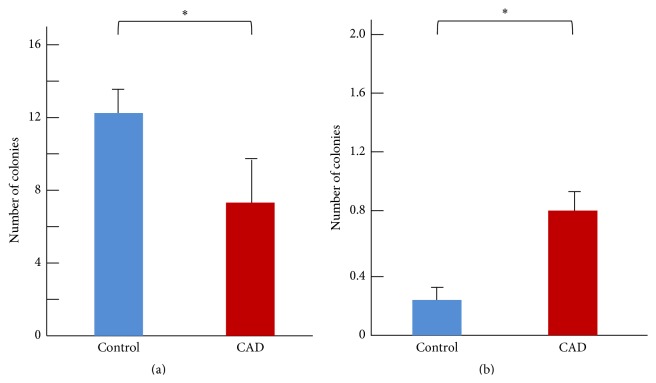
Mean number of observed early (a) and late (b) EPC colonies in control and CAD subjects. Early EPC colonies were counted on the 10th day of culture, and late EPC colonies were counted on the 28th day. ^*∗*^ *p* < 0.05.

There were significantly fewer early EPC colonies in the CAD group (7.2 ± 3.1/well) than in the control group (12.4 ± 1.4/well, *p* < 0.05). In contrast, the average number of late EPC colonies was higher in the CAD group (0.8 ± 0.2/well) than in the control group (0.25 ± 0.02/well, *p* < 0.05) (Figure 3). It was intriguing to examine early and late EPCs in subset with 12 control subjects who had coronary angiography. There were no differences in EPC behavior between these subjects and the remaining control subjects.

### 3.4. Evaluation of Gene Expression

**Figure 4:**
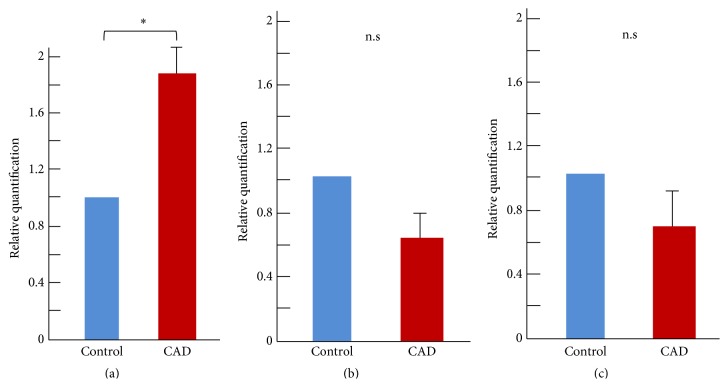
Evaluation of gene expression in early EPCs by qRT-PCR. Relative quantification of surface markers CD45 (a), CD31 (b), and KDR (c). ^*∗*^ *p* < 0.05.

**Figure 5:**
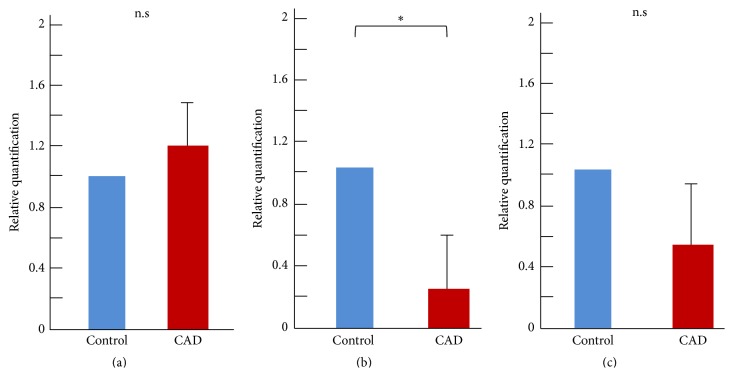
Evaluation of gene expression in late EPCs by qRT-PCR. Relative quantification of surface markers CD45 (a), CD31 (b), and KDR (c). ^*∗*^ *p* < 0.05.

Gene expression was analyzed in both types of EPC by qRT-PCR. The mRNAs of CD31, KDR, and CD45 were targeted; in general, CD31 and KDR are associated with endothelial lineage cells, and CD45 is associated with hematopoietic lineage cells [2]. In early EPCs, CD45 mRNA levels were significantly higher in the CAD patient group than in the control group (*p* < 0.05); however, CD31 and KDR mRNA levels tended to be higher in the control group than in the CAD patient group (Figure 4). In late EPCs, CD31 and KDR mRNA levels were higher in the control group than the CAD group (1.0 versus 0.22 and 1.0 versus 0.51, resp.). In particular, CD31 mRNA levels were significantly higher in the control group (*p* < 0.05). However, expression of CD45 did not differ between the control and CAD groups (1.0 versus 1.2) (Figure 5).

## 4. Discussion

In the present study, we demonstrate the following: (1) fewer early EPC colonies were produced in patients with CAD than in control subjects and higher late EPC colonies were found in patients with CAD similarly; (2) in early EPCs, levels of CD45 mRNA, which is a hematopoietic marker, were higher in CAD patients than in control subjects; (3) in late EPCs, the level of CD31 mRNA, which is an endothelial marker, was lower in CAD patients than in control subjects.

Previous studies have shown that the number of early EPC colonies decreases in patients exposed to cardiovascular risk factors. In the present study, we investigated the behavior of late EPC in addition to early EPC to find differences between early EPCs and late EPCs in CAD patients compared with control subjects, although it is still not clear whether the origin of early and late EPCs was the same or not. However, our results suggested at least that the quality, as well as the quantity, of EPCs was an important factor in endothelial repair and angiogenesis.

EPCs have been studied in conjunction with vascular tissue architecture, function, and disease states. Our results showed that both early and late EPCs presented surface markers, such as CD34 and KDR, taking up Ac-LDL and UEA-1 lectin, which were specific to endothelial cells [11, 12]. Differentiation from stem cells or progenitor cells to mature cells has been well studied in many systems and is characterized by a loss and gain of specific markers. EPCs exhibit various steps of differentiation from premature cells to mature cells. The proportion of expressed surface markers, including CD34, KDR, and CD45, changes from hematopoietic markers in immature cells to the endothelial markers in mature cells [12–14]. EPCs that originate from the hemangioblast in the bone marrow are released into peripheral circulation, and then they adhere to the tissues involved in repair of artery. Early EPCs emerge at an earlier period of culture than late EPCs; thus, the characteristics of early EPCs should be more similar to those of hematopoietic cells than late EPCs [11]. In accordance with these findings, our results showed that early EPCs were stained for CD45, a hematopoietic marker, confirming the hematopoietic character of early EPCs, which was similar to that of an immature cell.

It has been proposed that the number of EPC colonies is related to cardiovascular disease as with cerebrovascular disease and pedal artery disease. For example, several studies showed that the number of circulating early EPCs decreases in patients with diabetes, hypertension, and atherosclerosis [15–17]. In our study, the cell density of early EPCs in CAD patients was significantly lower than in control subjects. A decrease in circulating EPCs may disturb angiogenesis and is associated with a high risk of cardiovascular disease. Patients with risk factors for ischemic cardiovascular disease have fewer circulating EPCs, and that impaired migration of EPCs is negatively correlated with cardiovascular risk factors. In atherosclerotic diseases such as stroke and myocardial infarction, which occur as the complications in diabetes mellitus, hypertension, dyslipidemia, and so on, endothelial dysfunction is caused by several factors that reduce the viability and proliferation of EPCs and thereby increase apoptosis of EPCs or decrease levels of endothelial nitric oxide synthetase [18–21].

In this study, we focused on the relationship between the maturity of EPCs and CAD associated with risk factors like diabetes mellitus, hypertension, and dyslipidemia. Compared with control subjects, patients with CAD showed higher expression of the hematopoietic marker CD45 in early EPCs and lower expression of the endothelial marker CD31. This suggested that the peripheral circulating EPCs in CAD patients were more characteristic to immature EPCs than those in control subjects which consisted of at least subject without defined CAD. Furthermore, the gene expression profiles of both EPC types in CAD more closely resembled those of primitive endothelial cells. Therefore, evaluating the number of circulating immature EPCs, defined by surface markers such as CD45 and CD31, may provide additional insights into the mechanisms existing in the character of EPCs in CAD patients.

EPCs recruited to disease sites may contribute to repair and restore endothelial function. Therefore, it is important to evaluate not only the number of EPC cells related to endothelial formation and repair but also their quality. Specifically, it is an important factor whether EPCs are released into peripheral circulation before they mature into functional and proliferative cells. Further work will be required to examine how the maturity of EPCs affects the formation and repair of the endothelium in peripheral arteries, and perhaps this will need to include study of the tube formation. There was a significant difference in medications in comparing control and CAD subjects, thus impairing the present results. However, previous studies suggested that the impact of these medications on EPC behavior was minimized [6, 20].

There are some limitations in this study. First, the number of subjects and the number of colonies of late EPCs were relatively small; therefore, some group comparisons may lack the power to detect significant differences between variables. Second, the relationship between the EPC types and the severity of angiographic CAD was not specified. Particularly, it is of great interest to correlate severity of CAD and behavior of EPCs clarifying this clinic-pathological relation. Finally, no functional studies of endothelial function of the late EPC were performed, although it would be important to know if the late EPC functions were different when comparing control and CAD subjects. Further functional study such as migration, proliferation, and tube formation on Matrigel is necessary to confirm the functional differences.

## 5. Conclusions

We examined the behavior of early and late EPCs in order to understand the characteristics and roles of these EPCs in CAD. On the basis of our results, we suggest that changes in EPC colony count and gene expression of endothelial markers may have relation with development of CAD.

## Figures and Tables

**Table 1:** Clinical background of examined subjects.

Parameters	Control (*n* = 36)	CAD (*n* = 47)	*p* value
Male gender	22 (61%)	34 (72%)	n.s.
Age (years)	59 ± 14	67 ± 10	n.s.
Laboratory data			
WBC (/*μ*L)	6710 ± 2654	5992 ± 1770	n.s.
Neutrophil (%)	61 ± 12	63 ± 8	n.s.
Lymphocyte (%)	30 ± 2	26 ± 9	n.s.
MDA-LDL/LDL-chol.	1.05 ± 0.02	1.27 ± 0.11	n.s.
hs-CRP (mg/dL)	0.10 ± 0.1	0.22 ± 0.3	n.s.
HbA1c (%)	5.6 ± 0.5	5.9 ± 0.7	n.s.
Disease			
Diabetes mellitus	3 (8%)	14 (30%)	<0.05
Hypertension	3 (8%)	21 (45%)	<0.05
Hyperlipidemia	1 (3%)	20 (43%)	<0.05
Medication			
ARB or ACE-I	4 (11%)	22 (47%)	<0.05
CCB	0 (0%)	14 (30%)	<0.05
Statin	1 (3%)	20 (43%)	<0.05

ACE = angiotensin converting enzyme, ARB = angiotensin receptor blockage, CAD = coronary artery disease, and CCB = calcium channel blocker.
